# The Association of Acne Vulgaris and Disease Severity With Serum Amyloid A1 and Insulin Levels

**DOI:** 10.1111/jocd.70720

**Published:** 2026-03-12

**Authors:** Pelin Hizli, Seyma İçöz Aytaç, Özgür Baykan, Melisa Öklü, Fatma Arzu Kiliç

**Affiliations:** ^1^ Department of Dermatology, Faculty of Medicine Balikesir University Balikesir Turkiye; ^2^ Department of Dermatology Balikesir Atatürk City Hospital Balikesir Türkiye; ^3^ Department of Medical Biochemistry, Faculty of Medicine Balikesir University Balikesir Türkiye

**Keywords:** acne vulgaris, disease severity, inflammation, insulin, serum amyloid A1

## Abstract

**Background:**

Acne vulgaris is a chronic inflammatory disease involving multiple factors such as increased sebum production, follicular hyperkeratinization, microbial colonization, and inflammation. Serum amyloid A1 (SAA1), an acute phase protein, and insulin, a hormone linked to metabolic and inflammatory pathways, may play significant roles in acne pathogenesis.

**Objective:**

This study aimed to evaluate SAA1 and insulin levels in patients with acne vulgaris and to investigate their relationship with disease severity and scar formation.

**Methods:**

A total of 72 acne vulgaris patients [13 males, 59 females; median age 22 (19–34) years] and 66 age‐similar healthy controls [27 males, 39 females; median age 22 (18–38) years] were included. Acne severity was assessed using the Global Acne Grading System (GAGS), and scar severity was evaluated by the Global Scale for Acne Scar Severity. SAA1 and insulin levels were measured via ELISA from fasting blood samples. Additionally, anthropometric measurements and biochemical parameters were recorded.

**Results:**

A total of 138 participants were included, with 72 acne vulgaris patients and 66 healthy controls. The groups were age‐similar, though a higher female proportion was observed in the acne group. SAA1 levels were significantly higher in acne patients (*p* = 0.045), whereas insulin levels did not differ significantly (*p* = 0.902). LDL, triglycerides, and total cholesterol were significantly lower in the acne group (*p* = 0.003, *p* = 0.045, *p* = 0.023, respectively). SAA1 levels did not significantly correlate with acne severity (*p* = 0.052) or scar severity (*p* = 0.09). However, LDL and total cholesterol showed weak negative correlations with both acne severity and scar severity.

**Conclusion:**

Elevated SAA1 in acne vulgaris patients suggests that SAA1 may serve as a novel biomarker for assessing inflammation in acne. Further large‐scale studies are needed to explore therapeutic implications targeting inflammation.

## 1 Introduction

Acne vulgaris is a chronic inflammatory disease of the pilosebaceous unit, commonly seen especially during adolescence, and acne‐like lesions can occur at any age [1]. The pathogenesis of the disease involves factors such as increased sebum production by sebocytes, follicular hyper‐keratinization, colonization of *Cutibacterium acnes* (C. acnes), hormones, and inflammation. Acne lesions can present as comedones, papules, pustules, and nodules, which can significantly affect quality of life [2].

In recent years, the central role of inflammatory processes in acne pathogenesis has become increasingly understood. Various biomolecules such as inflammatory markers and adipokines have emerged as important players in this process [3]. In this context, serum amyloid A1 (SAA1) has attracted attention as one of the interesting biomarkers. It is an acute phase reactant synthesized in the liver in response to proinflammatory cytokines such as IL‐1, IL‐6, and TNF‐α, reflecting both systemic inflammation and local inflammatory responses [4]. SAA1 has been previously reported to facilitate the migration of neutrophils and monocytes to sites of inflammation, increase inflammatory cell infiltration, stimulate the expression of chemokines such as IL‐8, and attenuate anti‐inflammatory effects by binding to high‐density lipoproteins (HDL) [5]. In the bloodstream, SAA1 is typically bound to HDL at lower levels but it dissociates from these complexes when its concentration rises. Additionally, free SAA1 has been identified in sites of inflammation, suggesting a potential role in local inflammatory responses [5].

Insulin resistance, through hyperinsulinemia, can contribute to acne pathogenesis by increasing lipogenesis in sebocytes and causing follicular hyper‐keratinization [6]. The reciprocal interaction between insulin resistance and inflammation in acne vulgaris may be part of a complex mechanism that determines disease severity and prognosis.

This study aimed to investigate SAA1 and insulin levels in patients with acne vulgaris and to evaluate the relationship with disease severity and acne scars. The primary goal of the research was to reveal the potential roles of SAA1 and insulin levels in acne pathogenesis, thereby contributing to the existing literature in this field. The findings may allow for the development of new perspectives in the clinical evaluation and treatment approaches of acne patients. Additionally, it is intended to provide a scientific basis for future studies that will more comprehensively address the relationship between SAA1 levels, insulin resistance, and acne severity.

## 2 Materials and Methods

This prospective cohort study included 72 acne vulgaris patients aged between 18 and 45 years, admitted to the Dermatology Outpatient Clinic of our territory institution and received no systemic or topical treatment in the last 3 months, along with a control group of 66 healthy individuals matched for age without any skin disease. Both patient and control groups underwent blood analysis after at least 12 h fasting period. Individuals with malignancy, autoimmune disease, or chronic inflammatory disease were excluded from the study.

The study was approved by the Balıkesir University Clinical Research Ethics Committee (Ethics Committee Decision No: 2023/123), and written informed consent was obtained from all participants. Additionally, this research was supported by the Balıkesir University Scientific Research Projects (BAP) Coordination Unit (Project No: 2023/88).

Acne severity was assessed using the Global Acne Grading System (GAGS, 1997), and scar severity was evaluated with the Global Scale for Acne Scar Severity [7]. Demographic data, as well as height, weight, and body mass index (BMI) measurements, were recorded. Biochemical parameters including total cholesterol, HDL, LDL, triglycerides, and CRP levels were evaluated. SAA1 and insulin levels were measured from fasting morning serum samples using the ELISA method according to the manufacturer's protocol.

### 2.1 Statistical Analysis

The distribution of each variable was assessed using the Kolmogorov–Smirnov normality test. Only total cholesterol showed a normal distribution (*p* = 0.20). As mean and standard deviation are appropriate descriptive statistics for normally distributed data, total cholesterol values were expressed as mean ± standard deviation, and group comparisons were performed using the independent samples *t*‐test. All other variables did not follow a normal distribution (*p* < 0.05); therefore, these were expressed as median (minimum–maximum), as median values are less affected by skewness and outliers. Comparisons between groups for these variables were conducted using the Mann–Whitney *U* test. Comparison of gender distribution was performed using the chi‐square test. Correlations were performed employing Spearmen test. Moreover, a univariate analysis of variance was performed to assess the effects of acne status and gender, as well as their interaction, on serum amyloid levels. Statistical analyses were performed using SPSS version 30.0 for MacOS (SPSS Inc., Chicago, IL). A *p*‐value < 0.05 was considered statistically significant.

## 3 Results

A total of 138 individuals were eligible for this study. The acne vulgaris group consisted of 72 patients [13 males and 59 females, median age: 22 (19–34) years], and the control group consisted of 66 healthy individuals [27 males and 39 females, median age: 22 (18–38) years]. The groups were age‐similar; however, a female predominance was observed in the acne vulgaris group (82% vs. 59%, *p* = 0.003).

In the acne vulgaris group, the mean acne severity score according to the Global Acne Grading System was 17.6 ± 7.7 (interquartile range: 12–22). Scar assessment in the acne vulgaris group showed that 16 (22.2%) patients had a scar score of 1 (almost clear), 37 (51.4%) had a score of 2 (mild), 17 (23.6%) had a score of 3 (moderate), and 2 (2.8%) had a score of 4 (severe).

Table 1 presents the comparison of SAA1, BMI, and various blood parameters between the groups. There was no significant difference in CRP, HDL, and BMI between the acne vulgaris and control groups (*p* = 0.066, *p* = 0.355, and *p* = 0.113, respectively). However, the median SAA1 was significantly higher in the acne vulgaris group compared to the control group (*p* = 0.045). Moreover, median LDL, median triglyceride, and mean total cholesterol values were significantly lower in the acne vulgaris group compared to controls (*p* = 0.003, *p* = 0.045, and *p* = 0.023, respectively). No significant difference was found between the groups in terms of insulin levels (*p* = 0.902).

**TABLE 1:** Comparisons between the groups.

	Control group	Acne vulgaris group	*p*
Age (years)	22 (18–39)	22 (19–34)	0.287^a^
Serum amyloid A1	6 (2–26.4)	7.5 (2.5–35)	**0.045** ^a^
CRP	3 (3–7)	3 (3–23)	0.066^a^
HDL	56 (37–97)	56 (31–107)	0.355^a^
LDL	102.5 (48–446)	88.5 (46–155)	**0.003** ^a^
Triglyceride	88.5 (34–437)	74 (34–758)	**0.045** ^a^
Total Cholesterol	183.8 ± 34.7	170.7 ± 32.3	**0.023** ^b^
Insulin	6.73 (3.75–39.14)	6.12 (1.69–44.78)	0.902^a^
BMI	22.77 (17.37–50.41)	21.67 (11.57–37.55)	0.113^a^

*Note:* Bold *p* values (< 0.05) indicate a statistically significant difference between the groups, like elevated serum amyloid A levels and decreased LDL, triglyceride and total cholesterol levels in acne vulgaris group compared with the controls.

Abbreviations: BMI, body mass index; CRP, C‐reactive protein; HDL, high‐density lipoprotein; LDL, low‐density lipoprotein.

^a^ *p* value of Mann Whitney *U* test.

^b^ *p* value of independent samples *t*‐test.

In the univariate analysis of variance, the interaction between acne status and gender on SAA1 levels was not statistically significant (*p* = 0.417), indicating that gender did not modify the effect of acne on serum amyloid levels.

In Spearman correlation analysis, SAA1 levels were not significantly correlated with acne severity (*p* = 0.052, rho = 0.166) or scar severity (*p* = 0.09, rho = 0.145). However, a significant, negative but weak correlation was detected between acne severity and both total cholesterol (*p* = 0.021, rho = −0.196) and LDL (*p* < 0.001, rho = −0.28). Similarly, scar severity showed a significant, negative but weak correlation with both total cholesterol (*p* = 0.01, rho = −0.219) and LDL (*p* = 0.001, rho = −0.271).

## 4 Discussion

In this study, we found that SAA1 levels were significantly higher in patients with acne vulgaris compared to healthy individuals, underlining the key role of inflammation in acne pathogenesis [8]. SAA1 is a hepatic acute‐phase reactant induced by pro‐inflammatory cytokines such as IL‐1, IL‐6, and TNF‐α, and modulates lipid metabolism by displacing apolipoprotein A‐I from HDL particles [5]. This may partly explain the lower LDL and total cholesterol levels observed in the acne group.

Cutibacterium acnes can trigger sebocytic inflammation via TLR activation, and recent evidence highlights SAA1 as a marker of sebocyte response [9]. SAA1 also enhances neutrophil and monocyte chemotaxis and IL‐8 expression, reinforcing its pro‐inflammatory nature [5]. However, no significant correlation was found between SAA1 levels and acne or scar severity, suggesting that SAA1 reflects inflammation presence rather than clinical disease extent. Furthermore, the multifactorial pathogenesis of acne severity, influenced by hormonal, genetic, and environmental factors, may contribute to the weak relationship between SAA1 and severity.

The relationship between insulin resistance and acne vulgaris is a controversial topic in the literature. Although insulin influences sebocyte activity and androgen production [6, 10], this study found no difference in insulin levels between groups or correlation with severity, indicating individual variability and multifactorial influences on acne.

The effects of inflammation on lipid profiles have been widely discussed in the prior literature; particularly, high triglycerides, low HDL, and variable LDL levels are frequently reported in inflammatory processes [11]. However, how these parameters are affected in individuals with acne is not yet fully elucidated. In this study, a significant increase in SAA1 levels and a decrease in LDL levels were detected in individuals with acne vulgaris. SAA1 may contribute to this via formation of SAA‐LDL complexes, as reported in other inflammatory settings [11, 12, 13]. Kotani et al. also associated elevated SAA‐LDL levels with inflammation in obese smokers [12]. Similarly, the low LDL levels observed in our study group can be explained by increased inflammation and SAA levels. This suggests that acne has a systemic inflammatory component and may cause disruptions in lipoprotein metabolism. Additionally, it is known that HDL levels decrease, and its functions are impaired during inflammation. Therefore, these lipid changes observed in individuals with acne may point not only to cutaneous lesions but also to potential systemic effects.

In our study, median LDL, median triglyceride, and mean total cholesterol levels were found to be significantly lower in patients with acne vulgaris compared to healthy controls. Although both groups had lipid values within the generally accepted normal ranges, the relative decrease observed in the acne group was statistically significant. This may not indicate a pathological reduction, but it highlights a potential metabolic or behavioral difference between the groups. It is plausible that patients with acne vulgaris may consciously modify their diet to avoid foods perceived as acne‐triggering, particularly those rich in fats and cholesterol. Such behavioral factors could partly contribute to the observed differences in serum lipid levels, although the negative correlation with severity in our study suggests that this explanation alone is insufficient. Further studies with larger cohorts and controlled dietary data are warranted to confirm and clarify these observations.

On the other hand, Cai et al. reported SAA1 to bind to HDL, impairing HDL's anti‐inflammatory and cholesterol transport functions. The increase in SAA1 leads to loss of HDL function and impaired cholesterol reverse transport, contributing to an inflammation‐triggered dyslipidemia [14]. However, we found no significant difference in HDL between acne vulgaris and control groups. Nevertheless, the simultaneous observation of increased SAA1 levels and decreased LDL levels in individuals with acne supports this relationship, making sense in the context of SAA1's interaction with lipoproteins and the systemic effects of inflammation. Elevated SAA1 may bind LDL, leading to the formation of SAA1‐LDL complexes.

Strengths of the study include ELISA‐based SAA1 quantification, standardized severity assessments, and age‐similar controls. Limitations include the modest sample size, cross‐sectional design, lack of hormonal profiling, and indirect assessment of insulin resistance. Future research should incorporate larger, longitudinal cohorts with detailed inflammatory, hormonal, metabolic, and molecular analyses to better define the systemic impact of SAA1 in acne.

## 5 Conclusion

This study revealed that SAA1 levels were significantly higher in patients with acne vulgaris compared to the control group, supporting the critical role of inflammation in acne pathogenesis. Despite the elevation in SAA1 levels, no significant correlation was observed between SAA1 and disease severity or scar severity, suggesting that while SAA1 reflects the presence of systemic inflammation, it may not directly indicate clinical severity. Insulin levels did not differ significantly between groups, and no association was found between insulin levels and acne severity, indicating that insulin's role in acne pathogenesis may be complex and influenced by various individual and environmental factors. Additionally, decreased LDL and total cholesterol levels observed in acne patients, potentially linked to inflammation, highlight possible systemic metabolic alterations associated with acne. Further large‐scale, longitudinal studies are necessary to elucidate these relationships and explore the potential of targeting inflammation and metabolic pathways in acne management.

## Author Contributions

Conceptualization, methodology, software, writing – original draft, project administration: P.H. Validation: Ö.B., M.Ö., F.A.K. Formal analysis: P.H., Ş.İ.A. Investigation: P.H., Ş.İ.A., Ö.B. Resources: Ö.B., Ş.İ.A., M.Ö. Data curation: Ş.İ.A., Ö.B. Writing – review and editing: P.H., Ş.İ.A., F.A.K. Visualization: Ş.İ.A., M.Ö. Supervision: F.A.K.

## Funding

This work was supported by the Scientific Research Projects Coordination Unit of Balıkesir University (Project Number: 2023/088).

## Consent

All patients have participated in the study providing their own written informed consent.

## Conflicts of Interest

The authors declare no conflicts of interest.

## Data Availability

The data that support the findings of this study are available on request from the corresponding author. The data are not publicly available due to privacy or ethical restrictions.
